# The Fabrication of Ordered Bulk Heterojunction Solar Cell by Nanoimprinting Lithography Method Using Patterned Silk Fibroin Mold at Room Temperature

**DOI:** 10.1186/s11671-015-1194-7

**Published:** 2015-12-23

**Authors:** Guangzhu Ding, Qianqian Jin, Qing Chen, Zhijun Hu, Jieping Liu

**Affiliations:** College of Chemistry and Materials Science, Huaibei Normal University, Huaibei, 235000 China; Collaborative Innovation Center of Advanced Functional Composites of Anhui Province, Huaibei, 235000 China; Center for Soft Condensed Matter Physics and Interdisciplinary Research, Soochow University, Suzhou, 215123 China

**Keywords:** Nanoimprint lithography, Nanopattern, Ordered bulk heterojunction, Silk fibroin film

## Abstract

The performance of organic solar cell is greatly determined by the nanoscale heterojunction morphology, and finding a practical method to achieve advantageous nanostructure remains a challenge. We demonstrate here that ordered bulk heterojunction (OBHJ) solar cell can be fabricated assisted by a simple, cost-effective nanoimprinting lithography method using patterned silk fibroin film mold at room temperature. The P3HT nanogratings were achieved by nanoimprinting lithography (NIL) process, and phenyl-C61-butyric acid methyl ester (PCBM) was spin-coated on the top of P3HT nanogratings. The conducting capacity of P3HT nanograting film has little difference compared with the unimprinted film in the vertical direction, due to the same edge-on chain alignment. However, it can be found that the fabrication of OBHJ nanostructure using room temperature NIL technique with patterned silk fibroin mold is able to promote optical absorption, interfacial area, and bicontinuous pathway. Therefore, the ordered heterojunction morphology plays an important part in improving device performance due to efficient exciton diffusion, dissociation, and reducing charge recombination rate.

**Electronic supplementary material:**

The online version of this article (doi:10.1186/s11671-015-1194-7) contains supplementary material, which is available to authorized users.

## Background

The organic solar cells based on the bulk heterojunction (BHJ) have received considerable attention as an attractive alternative to silicon photovoltaic cell as they have achieved favorable characteristics, such as low cost, flexibility, and simple process [1, 2]. As long as there is light absorption, the excitons are generated, diffused, and dissociated at the interface between donor and acceptor materials and then transported to their respective electrodes, forming the external circuit [3]. Therefore, the performance of organic solar cell is greatly determined by the nanoscale heterojunction morphology within active layer. One of the ideal structures in active layer is to construct an ordered bulk heterojunction (OBHJ) morphology consisting of vertically bicontinuous and interdigitized heterojunction between donor and acceptor materials, to enable both efficient exciton separation and transport [4–6].

Despite OBHJ nanostructure morphology contributes to the solar cell performance and a comprehensive understanding of fundamental principle, finding a practical method to achieve this nanostructure remains a challenge to now. Several techniques, such as polymer nanowires, block copolymer, and nanoimprinting lithography (NIL), have been reported to fabricate OBHJ solar cell [7–9]. Among these techniques, thermal NIL is investigated as a promising method to define nanostructures due to its high resolution, effective cost, and simple process [10, 11]. NIL method is able to replicate the nanostructures defined on a hard mold into some soft materials, such as semiconducting polymers [12–19], ferroelectric polymers [20, 21], and proteins [22, 23]. Therefore, the control of nanoimprint mold is of great importance in the fabrication of OBHJ solar cell by NIL technique. Some traditional molds with high resolution over a large area, such as silicon mold or anodic aluminum oxide mold, are reported to apply; however, they are usually time-consuming, have complicated process, have simple fragility, crush or deformed easily, and do not to meet the need of commercial application. It is desired to seek a cost-effective and simple process method for mold fabrication.

Silk fibroin film from the Bombyx mori silkworm has attracted considerable interest owing to its biological, mechanical, and optical properties [24–27]. Silk fibroin film is able to be easily patterned by several techniques [22, 28] and has been applied to biocompatible and degradable electronic or photonic devices [29, 30]. In nanoimprinting process, the application of heat and pressure to a patterned silk fibroin mold, which is fabricated by control of the water content and beta sheet crystallinity within silk film, can be accomplished to transfer nanostructure to other soft materials [23]. Silk fibroin film was chosen for the nanoimprinting mold mainly due to its advantageous material properties as well as simple and inexpensive production process [31, 32]. The Bombyx mori silkworm which is used to produce patterned silk fibroin film comes from broad source and is cheap. Silk fibroin film is able to be easily patterned, and the fabrication process is simple and convenient, fully meet to the demand of large area production. Then, there is little interaction between silk film and conjugated polymer, for example, polymer P3HT, and thus, there is no need for special surface treating of patterned silk mold to facilitate template separation after nanoimprinting. Super stiffness and high modulus are present in the silk fibers, and it can guarantee to bear more pressure during NIL process and the three-dimensional size stability of nanostructure within silk film. Furthermore, compared to the conventional silicon or glass molds, the silk mold is not easy to be crushed and allows for direct conformal imprinting on curved surface, due to the flexibility of silk film material.

In addition, some conjugated polymer materials are easily oxidized and decomposed at elevated temperatures [33], potentially detrimental to the organic semiconducting device performance. To overcome the drawback of thermal NIL applied to conjugated polymers, room temperature NIL has been proposed to obtain desired nanostructures in conjugated polymer thin films [5, 12]. Therefore, it is significant at room temperature to produce the nanostructures of conjugated polymer by NIL method. The fabrication of OBHJ solar cell assisted by NIL technique with patterned silk fibroin film as nanoimprint mold, however, is rarely investigated, especially using the NIL process at room temperature.

In this study, we employ the patterned silk fibroin film as a template and room temperature NIL as a method to fabricate the active layer of OBHJ solar cell with P3HT as donor and phenyl-C61-butyric acid methyl ester (PCBM) as acceptor. We aim to report that the fabrication of OBHJ solar cell can be achieved by using the patterned silk fibroin film at room temperature. The influence of OBHJ active layer nanostructure fabricated by this convenient NIL method on the device performance of solar cell is investigated in details. Notably, the discussion of molecular orientation of polymer P3HT nanograting film is also investigated.

## Methods

Conjugated polymer P3HT (*M*_*w*_ 50,000 g mol^−1^; regioregularity 98 %) and PCBM (purity 99.5 %) were obtained from Rieke Metals Inc. and Solenne B. V. Co., respectively.

The purified silk fibroin solution was drop cast onto a clean silicon sheet (2 × 2 cm). The production of the silk fibroin solution had been carried out as the previously published protocols [34]. The patterned polydimethylsiloxane (PDMS) film was laid against the silk fibroin solution surface without exerting any pressure and dried for 24 h at room temperature. After removing the patterned PDMS film, the patterned silk film on the surface of silicon was treated with methanol solution (volume, 90 %) for about 5 h to induce β-sheet transition, leading to the patterned silk film water insoluble. The patterned silk fibroin films were subsequently dried for at least 24 h under vacuum.

The organic solar cells were fabricated with P3HT and PCBM as donor and acceptor materials, respectively. Indium tin oxide (ITO)-coated glass was washed with deionized water, ethanol, acetone, and isopropyl alcohol. After the glass was dried, PEDOT:PSS (about 30 nm thickness) was spin cast onto the ITO surface treated with ultraviolet ozone. Then, the whole substrates were annealed at 125 °C for 20 min in air. The P3HT thin films were obtained by spin coating (1600 rpm) from chlorobenzene solution (20 mg ml^−1^) onto PEDOT:PSS-coated ITO/glass substrate. After spin coating for 10 s, the polymer films were immediately transferred to a nanoimprinter system (Obducat, Eitre 3) and covered with patterned silk film. The nanoimprinting lithography process was performed under pressure (60 bar) at room temperature (23 °C) and held for 15 min. After the patterned silk film separated, the P3HT nanograting film was obtained. Then, PCBM in dichloromethane solution (10 mg ml^−1^) was spin-coated (800 rpm) onto the top of patterned P3HT film under ambient atmosphere for 60 s. For the contrast devices, the planar bulk heterojunction (PBHJ) solar cell was also fabricated by spin coating PCBM onto the unimprinted P3HT thin film. In the end, the devices were completed by evaporating a LiF layer (0.8 nm thickness) protected by aluminum electrode (100 nm thickness) at a base pressure of 4 × 10^−4^ Pa. The effective photovoltaic area was 12 mm^2^.

The morphology of samples was shown with scanning electron microscopy (SEM, Hitachi S-4800), with an operating voltage of 15 kV. Grazing incidence wide angle X-rays diffraction (GIWAXD) measurements were performed at the BL14B1 Beam line at the Shanghai Synchrotron Radiation Facility in China. The wavelength and the incident angle of the X-ray beam are 0.12398 nm and 0.18°, respectively. Data conversion to q space was obtained by calibration using LaB_6_ powder. The platinum/iridium-coated cantilevers (0.2 N/m force constant from nanosensors) were employed for the conducting atomic force microscopy (C-AFM) (MFP-3D-SA, Asylum Research) measurements, and the bias voltage between the ITO substrate and conducting cantilever (V_bias_) was 1.2 V under the atmosphere environment and at room temperature. UV absorption spectrum was performed using UV3600 spectrometer (Shimadzu) in the transmission geometry mode. Current-voltage characteristics of solar cells were measured under illumination of white light (100 mW cm^−2^) from a Hg-Xe lamp filtered by a Newport 81094 Air Mass Filter, using a Gwinstek SFG-1023 source meter. The external quantum efficiency (EQE) measurement was carried out with monochromatic light from Hg-Xe lamp (Newport 67005) and monochromator (Oriel, Cornerstone 260). The response was recorded as the voltage over a 50 Ω resistance, using a lock-in amplifier (Newport 70104 Merlin). All the measurement processes were carried out under ambient atmosphere and at room temperature.

## Results and Discussion

**Fig. 1:**
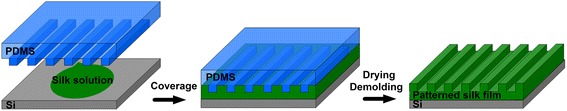
The fabrication process of patterned silk fibroin film mold

We demonstrate here the fabrication of OBHJ solar cell can be assisted by a simple, cost-effective nanoimprinting method with patterned silk fibroin film mold at room temperature. The control of patterned silk fibroin film mold is of great importance in the fabrication of OBHJ solar cell by NIL technique. The preparation process of patterned silk fibroin film, shown in Fig. 1, consists of the drop-casting of silk fibroin solution and the formation of silk nanostructure film replicated from patterned PDMS film. Silicon sheet is only employed as substrate. After removing the PDMS film, the patterned silk fibroin film on the surface of silicon is obtained conveniently. The thickness of silk fibroin film can be mainly controlled by its solution, and it is desired to have silicon substrate to sustain as the silk film is still too thin (about 100 nm). The water within silk solution enables the viscosity of the silk fibroin and is beneficial to the mobility of silk fibroin molecule, which contributes to fulfill the whole nanocavity of patterned PDMS film. Therefore, although the patterned PDMS film is laid against the silk fibroin solution surface without exerting any pressure or process, but the silk fibroin film is yet able to be patterned successfully after water volatilization. In addition, it is easy and simple to demold PDMS film from the silk fibroin surface due to the hydrophobic surface of PDMS film. The main aim of patterned silk film on the surface of silicon treated with methanol solution to induce β-sheet transition is to make the patterned silk film water insoluble and more stiff, facilitating the NIL process at atmospheric environment. Because the moisture within patterned silk fibroin mold inclines to damage the three-dimensional nanostructure of template and will greatly enhance the degradation of P3HT-based organic electronics, detriment to solar cell performance. Then, due to little interaction between silk film and conjugated polymer P3HT, there is no need for special surface treating of patterned silk mold to facilitate template separation after nanoimprinting. We note here that it is difficult to achieve the conjugated polymer nanostructure by room temperature NIL method using PDMS as imprinting mold directly, since the PDMS material is not only provided with limited stiffness but also easy to absorb the organic solvent and change into swell or deform, leading to be incapable of obtaining uniform nanostructure precisely. However, the patterned silk fibroin mold is able to overcome the above weakness of PDMS imprinting mold during the NIL process. Therefore, it is significant to fabricate the patterned silk fibroin film mold replicated from the patterned PDMS film by this simple and practical process.

It is noted that the patterned silk film on the surface of silicon is treated with methanol solution to make the patterned silk film water insoluble and can be used at atmospheric environment. Then, there is little interaction between silk film and conjugated polymer P3HT. Therefore, the silk mold can be reused again after the employment to fabricate the P3HT nanostructure film.

**Fig. 2:**
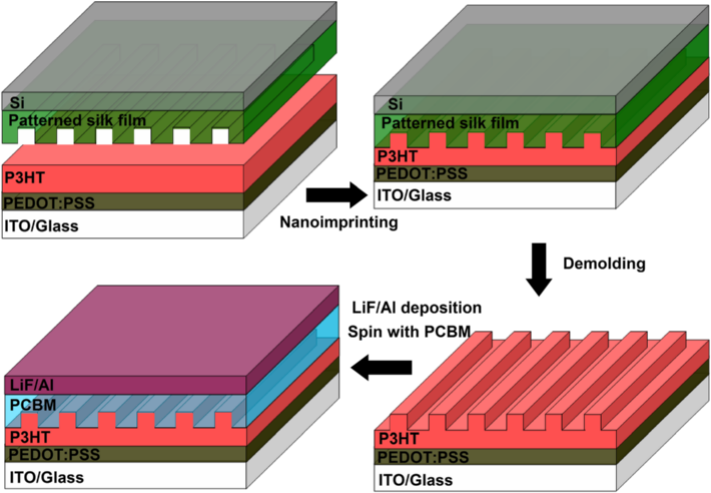
Procedure for the fabrication of OBHJ solar cell device

Then, the fabrication procedure of OBHJ solar cells assisted by room temperature NIL method with patterned silk fibroin mold is illustrated in Fig. 2, where P3HT and PCBM were chosen as donor and acceptor materials, respectively. Firstly, the polymer P3HT film is obtained by spin coating P3HT chlorobenzene solution onto PEDOT:PSS-coated ITO/glass substrate. Secondly, the P3HT nanogratings are achieved by pressing patterned silk fibroin mold against P3HT film at room temperature according to the NIL method process. Thirdly, PCBM material is spin-coated on the top of P3HT nanogratings, and the electrode is deposited onto the PCBM surface, to complete the fabrication process of OBHJ solar cell. The room temperature NIL process with patterned silk mold is able to guarantee highly reproducible and aligned P3HT nanogratings, due to the residual solvent assistant within the P3HT thin film (short spinning time) lowering the polymer viscosity [35] and the stiffness of patterned silk fibroin mold, which contribute to fulfill the whole nanocavity of silk fibroin template. In addition, dichloromethane is chosen as a solvent of PCBM material because it dissolves PCBM well but not P3HT material; therefore, the depositing PCBM layer process would not destroy the P3HT nanogratings and can ensure the nanostructure formation of OBHJ active layer for solar cell device. As a comparable device, the planar bulk heterojunction (PBHJ) solar cell is also fabricated by controlling PCBM material on the top of unimprinted P3HT thin film under the same preparation conditions. Therefore, it indicates that OBHJ solar cell is fabricated assisted by a simple, cost-effective nanoimprinting method using patterned silk fibroin film mold at room temperature.

**Fig. 3:**
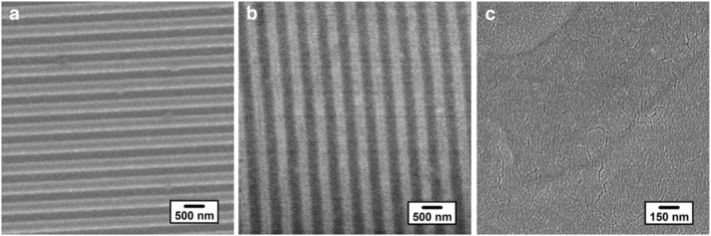
The top-down SEM images of template, P3HT film, and PCBM film surface. **a** The top-down SEM image of patterned silk fibroin template surface, *scale bar* = 500 nm; **b** the top-down SEM image of P3HT nanograting film surface fabricated by silk fibroin template (**a**), *scale bar* = 500 nm; **c** the top-down SEM image of PCBM surface deposited on the top of P3HT nanograting film, *scale bar* = 150 nm

In order to obtain a well-defined and stable active layer with bicontinuous pathways, fabrication of highly reproducible and well-controlled P3HT nanogratings is very important. Figure 3 shows the top-down SEM images of patterned silk fibroin template, P3HT nanogratings, and PCBM surface covered on the top of P3HT nanogratings film, respectively. The patterned silk fibroin mold, shown in Fig. 3a, consists of regular nanogratings bearing ~250-nm-wide trenches and a ~500 nm distance between the adjacent nanogratings (period). Under the effect of residual solvent, pressure, and the stiffness of the silk mold, the polymer P3HT flows into the nanocavities of patterned silk fibroin mold during NIL process at room temperature. After the silk mold demolding, the P3HT nanograting with ~250-nm width and ~500-nm pitch is obtained, as shown in Fig. 3b, which are faithfully transferred from the patterned silk mold. No collapses of P3HT nanogratings are observed, and this nanostructure is able to ensure the success in fabrication of OBHJ solar cells. It is critical for OBHJ solar cell to obtain a good interface between donor and acceptor and a higher coverage of P3HT nanogratings by PCBM, and inadequate coverage will result in poor cell performance [9]. Here, we carefully control the preparation conditions to form a continuous PCBM thin film, as shown in Fig. 3c, and the nanogratings are covered fully by PCBM with fewer defects. We note here that some fissures may be observed in the SEM image, which arises from the metal (Au) deposited onto the surface during the SEM measurement. It is noted that, according to the fabrication process of organic solar cell, there are five layers within the device in all. The thickness of P3HT film before the nanoimprinting lithography process is about 45 nm; however, the thickness of the residual layer after the nanoimprinting process is about 20 nm, and the depth of the P3HT nanograting is close to 50 nm. An additional file shows this in more detail (see Additional file 1). The thickness of other layers has been already indicated in the experimental method section. Therefore, the total thickness of the five layers within solar cell device is about 240 nm calculated from PEDOT:PSS layer to Al electrode layer.

**Fig. 4:**
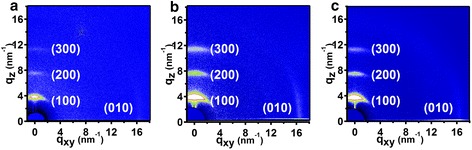
The two-dimensional (2D) GIWAXD images of P3HT film. **a** The 2D GIWAXD image of unimprinted P3HT film; **b** the 2D GIWAXD image of P3HT nanograting film with grating direction parallel to the direction of incident X-rays; **c** the 2D GIWAXD image of P3HT nanograting film with grating direction perpendicular to the direction of incident X-rays

As we know, the NIL method was able not only to fabricate nanostructure but also to induce the preferential polymer molecule orientation [18]. In our previous report, we had obtained the preferential face-on molecule alignment of polymer P3HT by solvent assistant room temperature nanoimprint lithography technique [36]. Thus, we also take the two-dimensional (2D) GIWAXD image to investigate the P3HT chain alignment, as shown in Fig. 4. In this paper, we also define the diffraction vector *q*_xy_ and *q*_z_ pointing parallel and vertical to the substrate plane, and the peaks at *q* = 3.8 nm^−1^ and *q* = 16.8 nm^−1^ correspond to the (100) plane and (010) plane reflections of polymer P3HT [36], respectively. To better investigate the molecular orientation for clarity, the GIWAXD measurement of P3HT nanograting films are carried out with nanograting direction parallel and perpendicular to the direction of incident X-rays, respectively. Apparently, only (*h*00) and (010) peaks appear in the *q*_z_ and *q*_xy_ direction, respectively, indicating that edge-on chain alignment dominates in the unimprinted film, as shown in Fig. 4a. Furthermore, the GIWAXD signals do not change for the P3HT nanograting films parallel or perpendicular to the direction of incident X-rays, as shown in Fig. 4b, c, indicating that chains alignment within nanograting film is also edge-on and displays a random distribution in the in-plane direction. The P3HT molecule alignment within nanostructure is different from our previous results [36], and this may be due to the difference in confinement dimension of P3HT rod-like crystals.

**Fig. 5:**
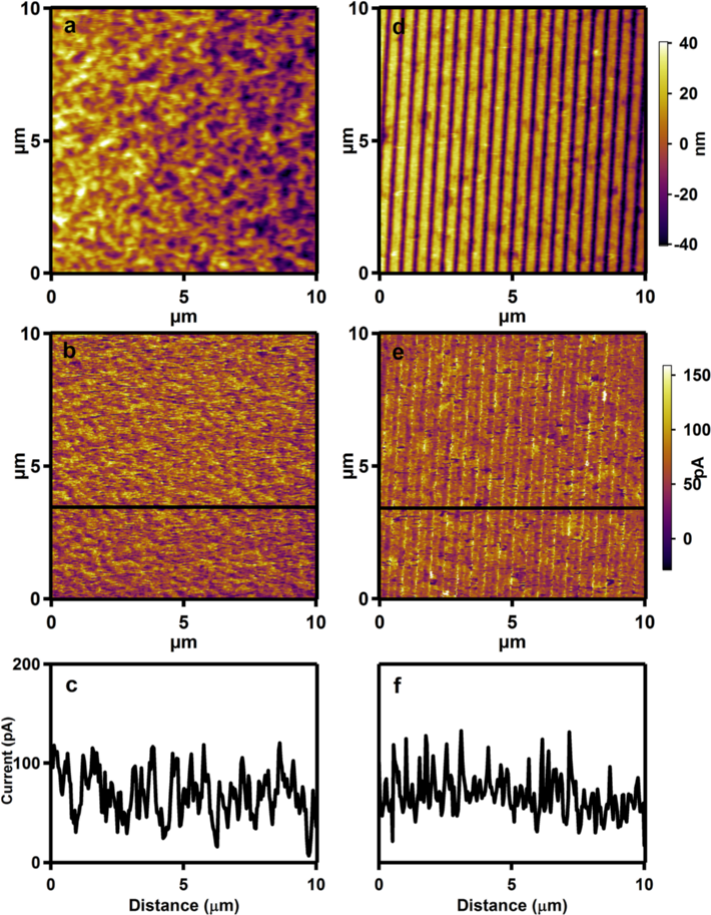
C-AFM height and current images of unimprinted and nanograting film. *Left column* C-AFM height image (**a**), current image (**b**), and cross-sectional current graph (**c**) of unimprinted P3HT thin film. *Right column*: C-AFM height image (**d**), current image (**e**), and cross-sectional graph (**f**) of P3HT nanograting film. The *black lines* in graphs (**b**) and (**e**) show the directions of cross-sectional images (**c**) and (**f**), respectively

In order to investigate the effect of NIL process with patterned silk fibroin mold on the conducting ability, the conducting performance of P3HT nanograting film is also investigated before fabricating the OBHJ solar cell. As we know, the conducting atomic force microscopy (C-AFM) is able to assess the conducting ability of conjugated polymer film in the vertical direction. The platinum/iridium-coated cantilevers are employed for C-AFM measurements, and the bias voltage between the ITO substrate and conducting cantilever (*V*_bias_) is applied to go through the sample film. Especially, the C-AFM measurement is able to offer morphology and conduct current of the sample film simultaneously. Figure 5 shows the typical C-AFM height, current images, and the corresponding cross-sectional current graphs of unimprinted P3HT film and P3HT nanograting film for comparison. The unimprinted film shows continual surface with slight roughness, and regular nanograting morphology is also investigated for the nanograting film, which is identical to the SEM image in Fig. 3. Then, comparing the two current images, we can see that the current value flowing through nanograting film is compared to the current of unimprinted film. Therefore, it can be inferred that the conducting capacity of P3HT nanograting film has little difference compared with the unimprinted film in the vertical direction. The similar conducting ability between the unimprinting and nanograting films may be mainly determined by their unanimous edge-on molecular orientation as discussed above [9]. In addition, the current value of unimprinted film is not uniform, meaning that slight difference of surface conductivity is present. This investigation mainly results from surface roughness of polymer film, and thus, current varies with position [9]. It is noted that there is a residual layer existing in the nanocavity of P3HT nanograting film, and thus, the conducting capability of P3HT nanograting film does not fluctuate largely between nanocavity and backbone within nanograting film. In all, it confirms that the NIL process with patterned silk fibroin mold at room temperature is not able to damage the charge transportation along the direction perpendicular to the substrate, i.e., the typical charge transportation direction of solar cell.

**Fig. 6:**
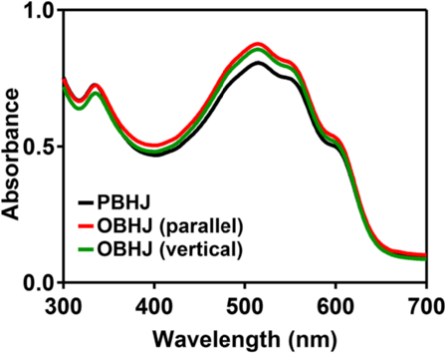
The UV absorbance spectra of PBHJ and OBHJ films. *Parallel* and *vertical* referred as measurements made with grating direction parallel and perpendicular to the direction of incident light

Then, we have fabricated the PBHJ and OBHJ active layer of solar cell. To further investigate the influence of P3HT nanogratings fabricated by the NIL technique with patterned silk fibroin mold on the optical absorption, the UV-vis absorption spectra of OBHJ and PBHJ films are measured, as shown in Fig. 6. The absorption peak centered at around 335 nm shows typical absorption of PCBM, and the locations do not alter for all the films obviously, which indicates the dimension of PCBM domains do not change largely [37]. The peaks at around 517, 552 (P3HT *π* − *π* * transitions), and 600 nm (P3HT inter-chain *π* − *π* interaction) are the typical absorption peaks of the ordered arrangement polymer P3HT to a certain extent [38]. It is particularly noteworthy that the absorption intensity of OBHJ film is enhanced after the NIL process with patterned silk fibroin mold and higher than the intensity of PBHJ film. As the amount of materials is not changed after the NIL process, the enhancement of the UV-vis absorption intensity can be attributed to light trapping and preferential molecular orientation [39]. The periodic nanostructure is able to enhance light trapping and thus increase the optical absorption by increasing the optical pass length. The preferential molecular orientation can also contribute to the enhancement of UV-vis absorption; however, unanimous edge-on molecular orientation is present in the P3HT unimprinting and nanograting films. Therefore, the absorption enhancement is mainly dependent on the light trapping of nangratings not the polymer molecule orientation. Furthermore, the absorption intensity of OBHJ film displays slight alteration between the nanograting line direction parallel and normal to incident light direction, showing that the optical absorption of OBHJ nanostructure is distributed evenly in in-plane direction. In addition, compared to the PBHJ film, no significant red-shift of P3HT molecule absorption peak is observed in the OBHJ film, which means that the conjugated length of P3HT molecules is not affected by the patterned process [40]. Therefore, it indicates that the fabrication of OBHJ nanostructure using NIL technique with silk fibroin mold at room temperature is able to promote the UV-vis absorption intensity, beneficial to the solar cell performance.

In addition, the periodic nanostructure is able to enhance light trapping and thus increases the optical absorption by increasing the optical pass length relatively. Therefore, the enhancement of UV-vis absorption intensity can be attributed to light trapping. Of course, the UV-vis absorption intensity can be promoted by simply increasing the thickness of P3HT layer. However, the same effect as the ordered bulk heterojunction solar cell cannot be obtained by the simply increasing the thickness of P3HT layer and without the nanostructure fabricated by the NIL method. There are three reasons. First of all, although the UV-vis absorption intensity is enhanced due to the increase of P3HT layer thickness, the efficiency of the excitons transporting to the corresponding electrodes is reduced obviously due to the increase of exciton transporting pathway, and thus the device current is also declined. Second, the enhancement of UV-vis absorption intensity is also limited only by increasing the thickness of P3HT layer. Third, the whole fabrication process of organic solar cell is complicated, and here, we aim to report that the fabrication of OBHJ solar cell can be achieved by using the patterned silk fibroin film at room temperature. The result indicates the periodic nanostructure fabricated by NIL method is able to enhance the UV-vis absorption intensity and it is not our goal to promote the UV-vis absorption intensity only. Therefore, it indicates that the fabrication of OBHJ nanostructure using NIL technique with silk fibroin mold at room temperature is able to promote the UV-vis absorption intensity, beneficial to the solar cell performance.

**Fig. 7:**
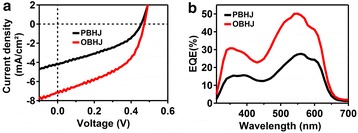
Device performance characteristics of solar cell. **a** *J-V* curves and (**b**) EQE curves of solar cells based on the PBHJ film and OBHJ film bearing P3HT nanogratings

To investigate the influence of nanograting active layer fabricated by the NIL technique using patterned silk fibroin mold on the device performance of solar cell, the photovoltaic responses of solar cells were obtained by measuring EQE curve and current density versus voltage (*J-V*) curve under illumination, as shown in Fig. 7a, b, respectively. The solar cell device based on PBHJ or OBHJ active layer is fabricated with a typical device structure (ITO/PEDOT:PSS/PBHJ or OBHJ/LiF/Al). The summary of device performance parameters of organic solar cells extracted from the *J-V* curves is presented in Table 1. All the measurements are carried out under an ambient atmosphere and at room temperature. The whole device performance parameters of OBHJ solar cell are preferential to that of PBHJ device obviously, especially in the *J*_SC,_ FF and power conversion efficiency (PCE).

**Table 1:** The device performance parameters of solar cells under the illumination

Active layer	*V*_OC_ (V)	*J*_SC_ (mA cm^-2^)	FF	PCE (%)
PBHJ	0.46	4.15	0.40	0.75
OBHJ	0.47	7.14	0.46	1.55

It indicates that the solar cell based on the OBHJ active layer film shows a significant improvement of *J*_SC_ (from 4.15 to 7.14 mA cm^−2^) due to the formation of bicontinuous transportation pathways within OBHJ film by the NIL process using silk mold. Considering about the mechanism of bulk heterojunction solar cell, the enhancement of *J*_SC_ of device based on OBHJ film may be determined by many factors. One reason for the circuit improvement is attributed to the enhanced light absorption intensity of P3HT nanograting within OBHJ active layer as discussed above, and thus, the device is able to absorb more photons to produce exciton. The second reason for the circuit increase may be attributed to the addition of interfacial area between the donor and acceptor materials. The aligned P3HT nanograting morphology within OBHJ active layer film fabricated by NIL process is able to effectively raise the interfacial area and in the end can efficiently increase the exciton dissociation efficiency, leading to the *J*_SC_ improvement. Third, with the fabrication of nanograting by NIL method, the exciton is able to more easily reach the interface between P3HT and PCBM due to the shorter pathway of exciton diffusion and the higher efficiency of exciton transportation, leading to facilitate the photocurrent generation. Therefore, the P3HT nanograting fabrication by NIL process using silk mold at room temperature significantly contributes to the *J*_SC_ improvement of OBHJ solar cell. Furthermore, the improving exciton dissociation efficiency due to the increased interfacial area between P3HT and PCBM is able to reduce the exciton recombination rate during exciton dissociation process, and thus it is inevitable to enhance the FF value of OBHJ solar cell.

As shown in Fig. 7b, the EQE curve of OBHJ solar cell is clearly enhanced compared to the curve of PBHJ solar cell, and the whole curves exhibit a broad response covering 300–700 nm wavelength. In addition, the EQE spectra of the devices resemble its absorption spectra of active layer used in the device, showing both components (P3HT and PCBM) contributing to the photocurrent. Although the molecular orientation and vertical conducting property of nanograting P3HT film are compared to unimprinted film, the ordered heterojunction morphology plays an important part in improving device performance due to optical absorption enhancement, interfacial area increase and bicontinuous pathway. The aligned P3HT nanograting structure of active layer can contribute to exciton diffusion and dissociation and reduction of charge recombination rate. Therefore, *J*_SC,_ FF and PCE of solar cell based on OBHJ film show a larger value compared to PBHJ solar cell. In all, these improved performances firmly confirm that the NIL method using patterned silk fibroin mold at room temperature is an effective technique to fabricate an ideal active layer bearing bicontinuous pathways and significantly improve the solar cell performance.

It indicates that although the solar cell fabricated by NIL process using patterned silk fibroin mold shows two times PCE than that of controlled PBHJ device, the efficiency does not show even higher value anticipated. However, in this study, we aim to report that the fabrication of OBHJ solar cell can be achieved by NIL process using the patterned silk fibroin film at room temperature and to investigate the influence of OBHJ active layer nanostructure fabricated by this convenient NIL method on the device performance of solar cell. Therefore, the absolute value seeking of solar cell efficiency is not our target. The limited efficiency of solar cell based on OBHJ active layer film may be determined by these factors: (a) the feature size with ~250 nm is much larger than the typical exciton diffusion length (10~20 nm) and limits more efficient excitons dissociation; (b) the fabrication process is performed under room temperature, and there is no any thermal history; thus the crystallinity of P3HT material is finite, which may affect the charge transport; (c) the device fabrication and characteristics testing process are all carried out under ambient atmosphere and not protected by any inert gas or in the glove box, which may reduce the device performance.

## Conclusions

In summary, we demonstrated here that solar cell device based on OBHJ film can be fabricated by a simple, cost-effective nanoimprinting lithography method using nanopatterned silk fibroin mold at room temperature. The preparation of patterned silk fibroin mold, consisting of the drop-casting of silk fibroin solution and the formation of silk nanostructure film replicated from patterned PDMS film, was a simple and practical process. P3HT and PCBM were chosen as donor and acceptor materials, respectively. The P3HT nanogratings were achieved by pressing patterned silk fibroin mold against P3HT film at room temperature, and PCBM was spin-coated on the top of P3HT nanogratings. It indicated that edge-on chain alignment dominated in P3HT nanograting film, which may be due to unconfinement of P3HT rod-like crystals. Therefore, it showed that the NIL process with patterned silk fibroin mold at room temperature was not able to damage the charge transportation along the direction perpendicular to the substrate. However, the fabrication of OBHJ nanostructure was able to promote the UV-vis absorption intensity, beneficial to the solar cell performance. Furthermore, the ordered heterojunction morphology played an important part in improving device performance due to optical absorption enhancement, interfacial area increase, and bicontinuous pathway. Therefore, *J*_SC,_ FF and PCE of solar cell based on OBHJ film were enhanced because the aligned P3HT nanograting structure of active layer contributed to exciton diffusion and dissociation and reducing charge recombination rate.

## Additional File

Additional file 1:
**The cross-section SEM images of layers within solar cell before (a) and after (b) the depositing of PCBM and LiF/Al layers on the top of P3HT nanograting film.** The cross-section SEM images of layers within solar cell are shown to offer some relevant data about the thickness of each layer in the solar cell. (DOC 235 kb)
