# Supplementary material for: The Fabrication of Ordered Bulk Heterojunction Solar Cell by Nanoimprinting Lithography Method Using Patterned Silk Fibroin Mold at Room Temperature
Source: Nanoscale Res Lett. 2015 Dec 23;10:491. doi: 10.1186/s11671-015-1194-7 (PMC4689722; doi:10.1186/s11671-015-1194-7)
Supplement: Additional file 1: — The cross-section SEM images of layers within solar cell before (a) and after (b) the depositing of PCBM and LiF/Al layers on the top of P3HT nanograting film. The cross-section SEM images of layers within solar cell are shown to offer some relevant data about the thickness of each layer in the solar cell. (DOC 235 kb) [file 11671_2015_1194_MOESM1_ESM.doc]

**Additional file 1**


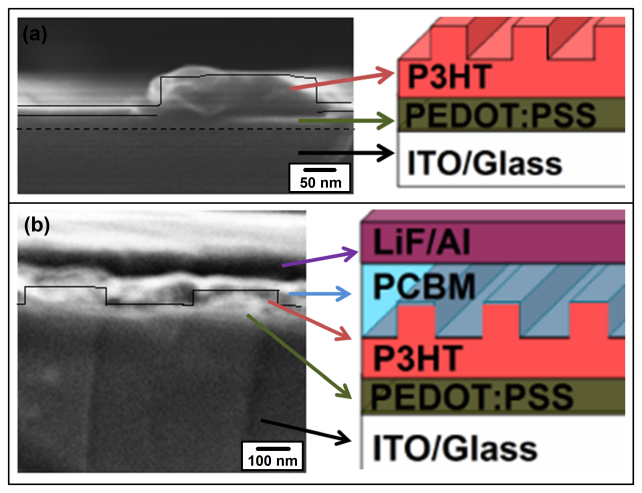


**The cross-section SEM images of layers within solar cell before (a) and after (b) the depositing of PCBM and LiF/Al layers on the top of P3HT nanograting film.**

As shown in SEM image, it indicates that there are five layers within the device in all. The thickness of the residual layer after the nanoimprinting process is about 20 nm and the depth of the P3HT nanograting is close to 50 nm. In addition, the thickness of PEDOT:PSS and LiF/Al layers are close to 30 nm and 100 nm respectively. The thickness of PCBM layer on the top of P3HT nanograting line is about 40 nm. Therefore, the total thickness of five layer within solar cell device is about 240 nm calculated from PEDOT:PSS layer to Al electrode layer.
